# Training trial staff in the use of a mixed method assessment of understanding tool in HIV vaccine trials

**DOI:** 10.1186/1742-4690-9-S2-P237

**Published:** 2012-09-13

**Authors:** J Mbogua, T Mebrahtu, G Lindegger, W Sabrina, O Anzala, G Mutua, J Mpendo, E Karita, S Singh

**Affiliations:** 1 International AIDS Vaccine Initiative, Nairobi, Kenya; 2 HIV AIDS Vaccine Ethics Group (HAVEG), KwaZulu Natal, South Africa; 3 Kenya AIDS Vaccine Initiative (KAVI), Nairobi, Kenya; 4 Uganda Virus Research Institute (UVRI), Entebbe, Uganda; 5 Projet San Francisco, Kigali, Rwanda

## Background

Volunteer understanding of trial concepts is a critical part of informed consent in clinical trials. Standard practice is to use closed-ended true/false forms to assess understanding; however such tools may overestimate understanding and most likely measure memory only. Open ended tools, such as scenarios, may be preferable. Following pilot studies that compared various methods of assessing understanding, a mixed method tool was developed by HAVEG, IAVI and partners. The tool employs both True/False questions for simpler concepts and open-ended scenarios for complex concepts.

## Methods

Trainings on use and implementation of the mixed method tool were conducted with doctors and counselors from IAVI partner research centers in Kenya, Uganda and Rwanda. Their understanding of all informed consent concepts was assessed, and practice sessions with past volunteers and other community staff with similar profiles to actual trial volunteers were conducted.

## Results

Initially the training participants did not see the benefit of the new tool, and expressed reservations about cost, skill, screen outs and time required to implement it.

However, following exposure to the tool, the participants felt the tool improves interaction between staff and volunteers, better evaluates understanding and identifies challenging concepts. They reported that scenarios bring to light unfounded assumptions about volunteer understanding or lack thereof.

It also became apparent that the mixed methods tool requires staff to have much better understanding of trial concepts themselves.

“Volunteers” also seemed to prefer the tool as it offered them an opportunity to demonstrate their understanding.

## Conclusion

Training significantly increases support for the mixed method tool reflecting the importance capacity building. Further, staff responses suggest mixed method tools of assessing understanding, not only provide better assessment of concepts, but may be preferred by trial staff and volunteers.

